# Cyclosporine A Induces Apoptotic and Autophagic Cell Death in Rat Pituitary GH3 Cells

**DOI:** 10.1371/journal.pone.0108981

**Published:** 2014-10-09

**Authors:** Han Sung Kim, Seung-Il Choi, Eui-Bae Jeung, Yeong-Min Yoo

**Affiliations:** 1 Department of Biomedical Engineering, College of Health Science, Yonsei University, Wonju, Gangwon-do, Republic of Korea; 2 Cornea Dystrophy Research Institute and Department of Ophthalmology, Yonsei University College of Medicine, Seoul, Republic of Korea; 3 Laboratory of Veterinary Biochemistry and Molecular Biology, College of Veterinary Medicine, Chungbuk National University, Cheongju, Republic of Korea; Swedish Medical Center, United States of America

## Abstract

Cyclosporine A (CsA) is a powerful immunosuppressive drug with side effects including the development of chronic nephrotoxicity. In this study, we investigated CsA treatment induced apoptotic and autophagic cell death in pituitary GH3 cells. CsA treatment (0.1 to 10 µM) decreased survival of GH3 cells in a dose-dependent manner. Cell viability decreased significantly with increasing CsA concentrations largely due to an increase in apoptosis, while cell death rates due to autophagy altered only slightly. Several molecular and morphological features correlated with cell death through these distinct pathways. At concentrations ranging from 1.0 to 10 µM, CsA induced a dose-dependent increase in expression of the autophagy markers LC3-I and LC3-II. Immunofluorescence staining revealed markedly increased levels of both LC3 and lysosomal-associated membrane protein 2 (Lamp2), indicating increases in autophagosomes. At the same CsA doses, apoptotic cell death was apparent as indicated by nuclear and DNA fragmentation and increased p53 expression. In apoptotic or autophagic cells, p-ERK levels were highest at 1.0 µM CsA compared to control or other doses. In contrast, Bax levels in both types of cell death were increased in a dose-dependent manner, while Bcl-2 levels showed dose-dependent augmentation in autophagy and were decreased in apoptosis. Manganese superoxide dismutase (Mn-SOD) showed a similar dose-dependent reduction in cells undergoing apoptosis, while levels of the intracellular calcium ion exchange maker calbindin-D9k were decreased in apoptosis (1.0 to 5 µM CsA), but unchanged in autophagy. In conclusion, these results suggest that CsA induction of apoptotic or autophagic cell death in rat pituitary GH3 cells depends on the relative expression of factors and correlates with Bcl-2 and Mn-SOD levels.

## Introduction

Programmed cell death (PCD) can be classified as either type I or type II PCD, based on distinctive morphological and biochemical characteristics. Type I PCD, or apoptosis, is characterized by blebbing, changes to the cell membrane such as loss of membrane asymmetry and attachment, cell shrinkage, nuclear fragmentation, chromatin condensation, and the formation of apoptotic bodies (apoptosomes) [1]. In contrast, type II PCD, or autophagy, is marked by extensive autophagic degradation of intracellular organelles, resulting in lysosome-associated cytoplasmic vacuolation/autophagosome formation [2]. Microtubule-associated protein 1 light chain 3 (LC3) is a marker for the autophagic process during which it is converted from the cytosolic form, LC3-I, to LC3-II, a modified form that is localized to autophagosomal membranes [3].

Currently, there is no known overlap in the pathways that modulate autophagic and apoptotic cell death [4], [5]. However, apoptotic and autophagic processes may functionally coordinate in three ways: both apoptosis and autophagy can cooperate to induce cell death; autophagy can act as an antagonist to block apoptotic cell death; and autophagy can act as a precursor or even initiator of apoptosis. Several specific examples of coordination between autophagy and apoptosis have been documented. The proapoptotic molecule TRAIL mediates autophagy [6]. In addition, growth factor deprivation induces an autophagic cell death, which can be inhibited by the anti-apoptotic factor Bcl-2 [7]. These data suggest that autophagic cell death may be induced by HSpin1, a transmembrane protein that interacts with Bcl-2/Bcl-xL [8].

Cyclosporine A (CsA) was first approved by the United States Food and Drug Administration in the early 1980s, and has been used significantly in prophylactic anti-rejection therapy for patients receiving allogeneic transplants (kidney, liver, and heart) for over two decades [8], [9]. However, several side effects of CsA have been reported in both transplant and non-transplant (i.e., individuals with autoimmune disorders) patients, including nephrotoxicity, hepatotoxicity, neurotoxicity, hypertension, dyslipidemia, gingival hyperplasia, hypertrichosis, malignancies, and an increased risk of cardiovascular events [10], [11]. Recent reports demonstrate that CsA induces autophagy *in vitro* in human tubular cells and *in vivo* in rat kidneys, and it has been suggested that autophagy serves as a protective mechanism against cyclosporine toxicity [12]–[14]. Importantly, it has been shown that cyclosporine-induced autophagy is triggered by endoplasmic reticulum (ER) stress [15]. Other reports show that CsA can induce apoptosis in human proximal tubular cells *in vitro* [16] and induces apoptosis via prolonged ER stress in an experimental model of chronic nephropathy [17].

In this study, we investigated whether CsA induces apoptotic and/or autophagic cell death in rat pituitary GH3 cells and correlated the levels of several characteristic molecular markers of these two pathways with cell death outcomes.

## Results

### Cell viability and LC3 expression following CsA treatment

**Figure 1:**
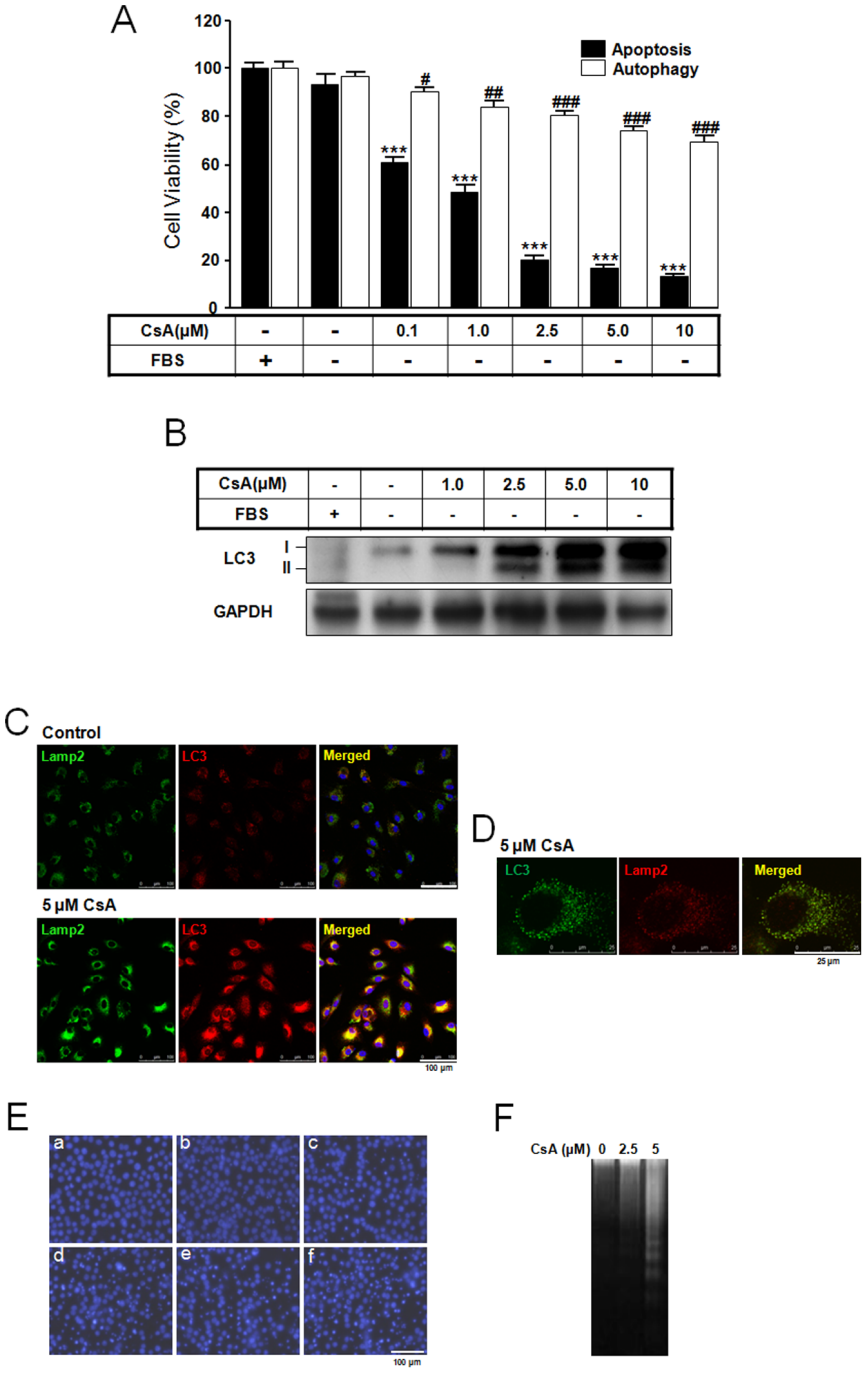
Cell viability and LC3 expression following CsA treatment. GH3 cells were incubated in DMEM with and without 10% fetal bovine serum in the presence or absence of CsA (0 to 10 µM) for 10 h. Cell survival was determined using Cell Counting Kit-8 (A) and LC3 expression was determined by Western blotting (B) as described in Materials and Methods. Immunofluorescence staining of LC3 and Lamp2 (C, D), DAPI staining (E), and DNA fragmentation (F) were captured as described in Materials and Methods. E: a, with FBS; b, without FBS; c, 1.0 µM CsA; d, 2.5 µM CsA; e, 5.0 µM CsA; f, 10 µM CsA. Scale bars: C, 100 µm; D, 25 µm; E, 100 µm. ***p<0.001 vs. serum treatment. ^#^ *p*<0.05, ^##^ *p*<0.01, ^###^ *p*<0.001 vs. serum treatment.

**Figure 2:**
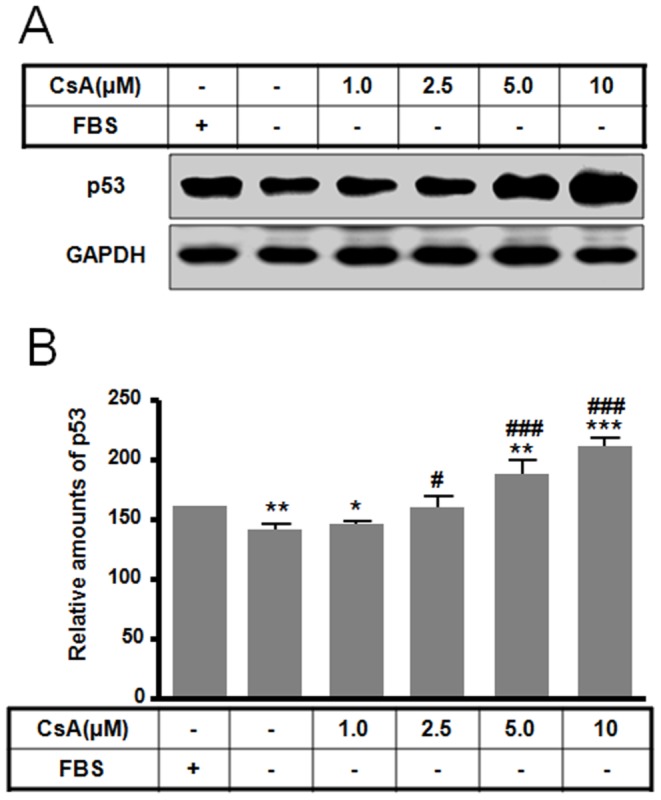
Effect of CsA-mediated apoptotic cell death on p53 expression levels. GH3 cells were incubated in DMEM with or without 10% fetal bovine serum in the presence or absence of CsA (0 to 10 µM) for 10 h. p53 expression (A) was determined by Western blotting and the relative amount (B) was calculated as described in the Materials and Methods. *p<0.05, **p<0.01 vs. serum treatment. ^#^ *p*<0.05, ^###^ *p*<0.001 vs. no serum treatment.

Many *in vivo* and *in vitro* studies have shown that CsA induces either autophagy or apoptosis [12]–[14], [16], [17]. To further these studies and examine how CsA concentration alters cell death pathway fate, we investigated CsA treatment for 10 h at different CsA doses. For autophagy study, polyethyleneimine was coated on culture dish. CsA treatment induced apoptotic and autophagic cell death with distinct toxicities in rat pituitary GH3 cells. CsA treatment (0.1 to 10 µM) decreased survival of rat pituitary GH3 cells in a dose-dependent manner. Apoptosis resulted in an 87% decrease in cell viability following treatment with 10 µM CsA, whereas autophagy resulted in a 30% reduction in cell viability at the same dose (Fig. 1A). At concentrations ranging from 1.0 to 10 µM, CsA induced a dose-dependent increase in the expression of LC3-I and LC3-II (Fig. 1B). At 5 µM CsA, immunofluorescence staining were performed to detect the co-localization of LC3 and lysosomal-associated membrane protein 2 (Lamp2). The increased LC3-positive granules or puncta were co-localized with the increased Lamp2, indicating increases in autophagosomes (Fig. 1C, D). Together, these data indicate that CsA induces autophagy in rat pituitary GH3 cells. In parallel, some CsA concentrations also induced apoptotic cell death in a dose-dependent manner as assayed by nuclear fragmentation with DAPI staining (Fig. 1E). In particular, treatment with 2.5 to 10 µM CsA induced clear nuclear and DNA fragmentation (Fig. 1E, F) and an increase in p53 expression (Fig. 2) compared to treatment with or without serum. These data indicate that 1.0 to 10 µM CsA can induce dose-dependent autophagy and apoptosis in GH3 cells.

### The differences of molecular levels between apoptosis and autophagy

**Figure 3:**
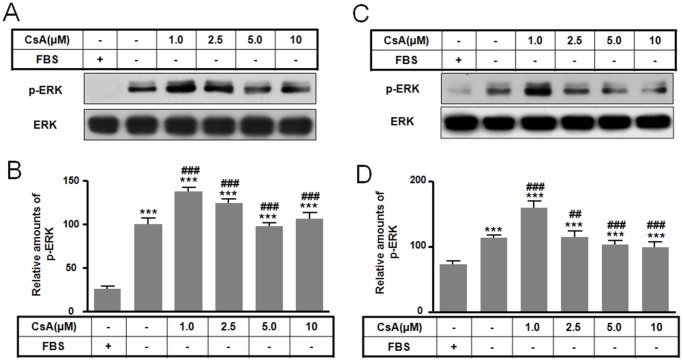
Effect of CsA-mediated autophagic and apoptotic cell death on p-ERK levels. GH3 cells were incubated in DMEM with or without 10% fetal bovine serum in the presence or absence of CsA (0 to 10 µM) for 10 h. Levels of p-ERK for autophagy (A) and apoptosis (C) were determined by Western blotting and the relative amount of p-ERK for autophagy (B) and apoptosis (D) was calculated as described in the Materials and Methods. ***p<0.01 vs. serum treatment. ^##^ *p*<0.01, ^###^ *p*<0.001 vs. no serum treatment.

**Figure 4:**
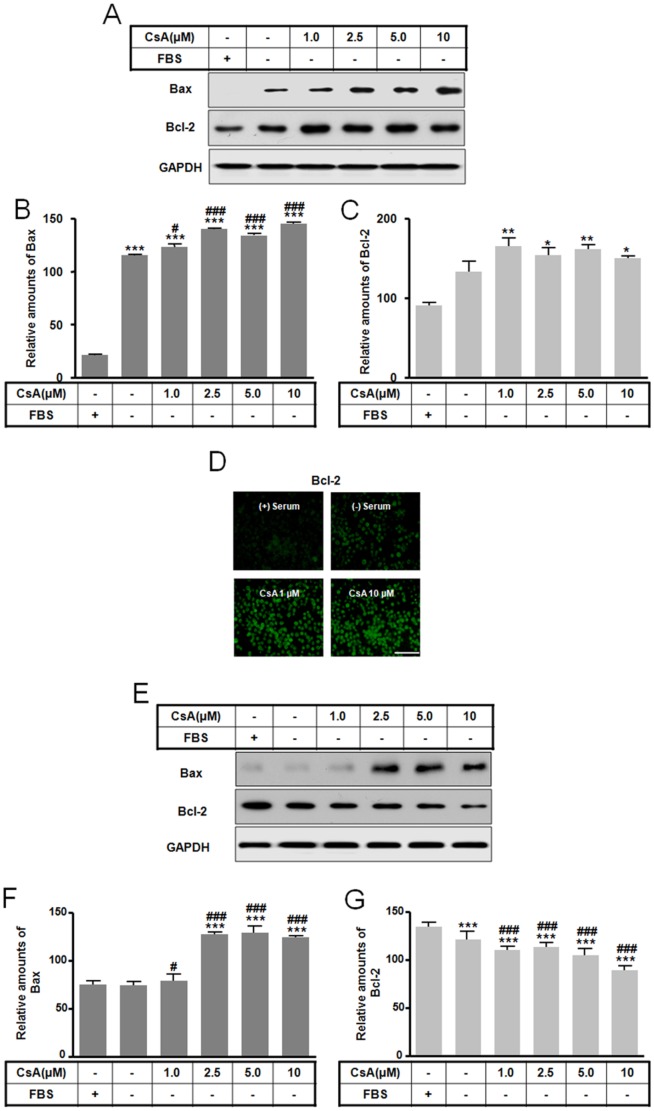
Effect of CsA-mediated autophagic and apoptotic cell death on Bax and Bcl-2 levels. GH3 cells were incubated in DMEM with or without 10% fetal bovine serum in the presence or absence of CsA (0 to 10 µM) for 10 h. Levels of Bax and Bcl-2 for autophagy (A) and apoptosis (E) were determined by Western blotting and the relative amount of Bax and Bcl-2 for autophagy (B, C) and apoptosis (F, G) was calculated as described in the Materials and Methods. Bcl-2 was imaged on an OLYMPUS DP controller and manager using an inverted microscope (D). *p<0.05, **p<0.01, ***p<0.001 vs. serum treatment. ^#^ *p*<0.05, ^###^ *p*<0.001 vs. no serum treatment. Scale bar is 100 µm.

Levels of several molecules implicated in PCD pathways were examined in cells undergoing CsA induced apoptosis or autophagy. In both apoptosis and autophagy, p-ERK levels were highest following treatment with 1.0 µM CsA and decreased following 2.5 to 10 µM CsA treatment (Fig. 3). In contrast, Bax levels were altered in a dose-dependent fashion that varied with the cell death pathway, showing an increase in autophagy (Fig. 4A, B) and apoptosis (Fig. 4E, F). These changes in p-ERK and Bax levels demonstrate that CsA toxicity can influence survival of GH3 cells depending on the CsA dose. Bcl-2 levels increased during autophagy following treatment with 1.0 and 10 µM CsA as measured by western blot (Fig. 4A, C) and immunofluorescence (Fig. 4D), while Bcl-2 levels decreased during apoptosis (Fig. 4E, G). These data suggest that Bcl-2 protein expression may result in difference between apoptosis and autophagy by CsA.

### The differences of Cu/Zn- and Mn-SOD between apoptosis and autophagy

**Figure 5:**
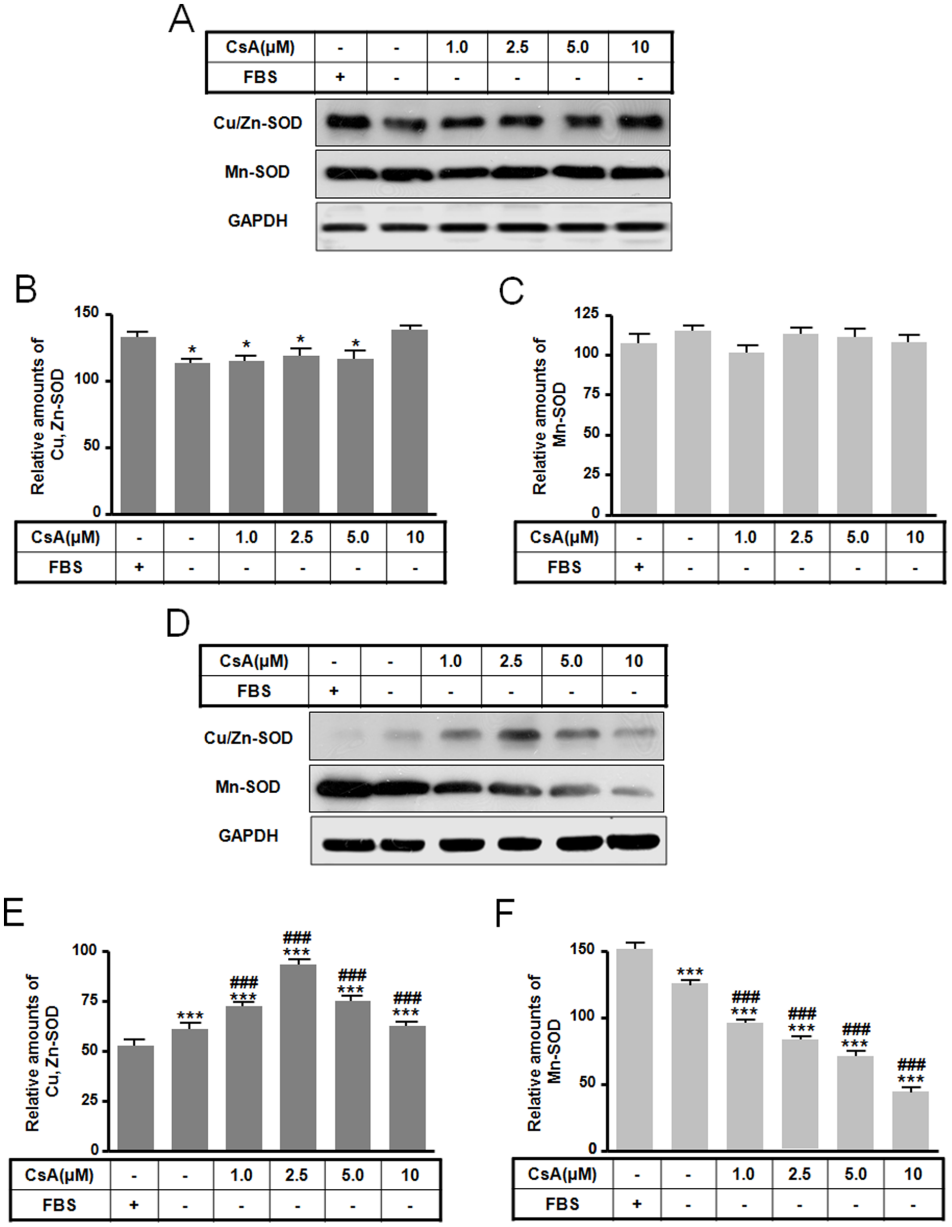
Effect of CsA-mediated autophagic and apoptotic death on Cu/Zn- and Mn-SOD levels. GH3 cells were incubated in DMEM with or without 10% fetal bovine serum in the presence or absence of CsA (0 to 10 µM) for 10 h. Cu/Zn- and Mn-SOD levels for autophagy (A) and apoptosis (D) were determined by Western blotting and the relative amount for autophagy (B, C) and apoptosis (E, F) was calculated as described in the Materials and Methods. *p<0.05, ***p<0.001 vs. serum treatment. ^###^ *p*<0.001 vs. no serum treatment.

CsA-induced nephrotoxicity may result from oxidative stress, and correspondingly, antioxidant enzymes, including SOD, catalase, and glutathione peroxidase, were found to be reduced in CsA related toxicity [18]. To examine these alterations in relationship to CsA-induced cell death, we assayed the levels of Cu/Zn- and Mn-SOD. CsA-mediated autophagy resulted in slightly lower levels of Cu/Zn-SOD expression, while Mn-SOD expression was relatively unchanged (Fig. 5A–C). In apoptotic cells, Cu/Zn-SOD expression was increased following 2.5 µM CsA treatment and higher doses decreased levels to those observed in the serum free condition (Fig. 5D, E). Mn-SOD expression showed a dose-dependent reduction (Fig. 5D, F). These results suggest that a decline in Mn-SOD levels may induce CsA-mediated apoptotic cell death in GH3 cells.

### The difference of calbindin-D9k between apoptosis and autophagy

**Figure 6:**
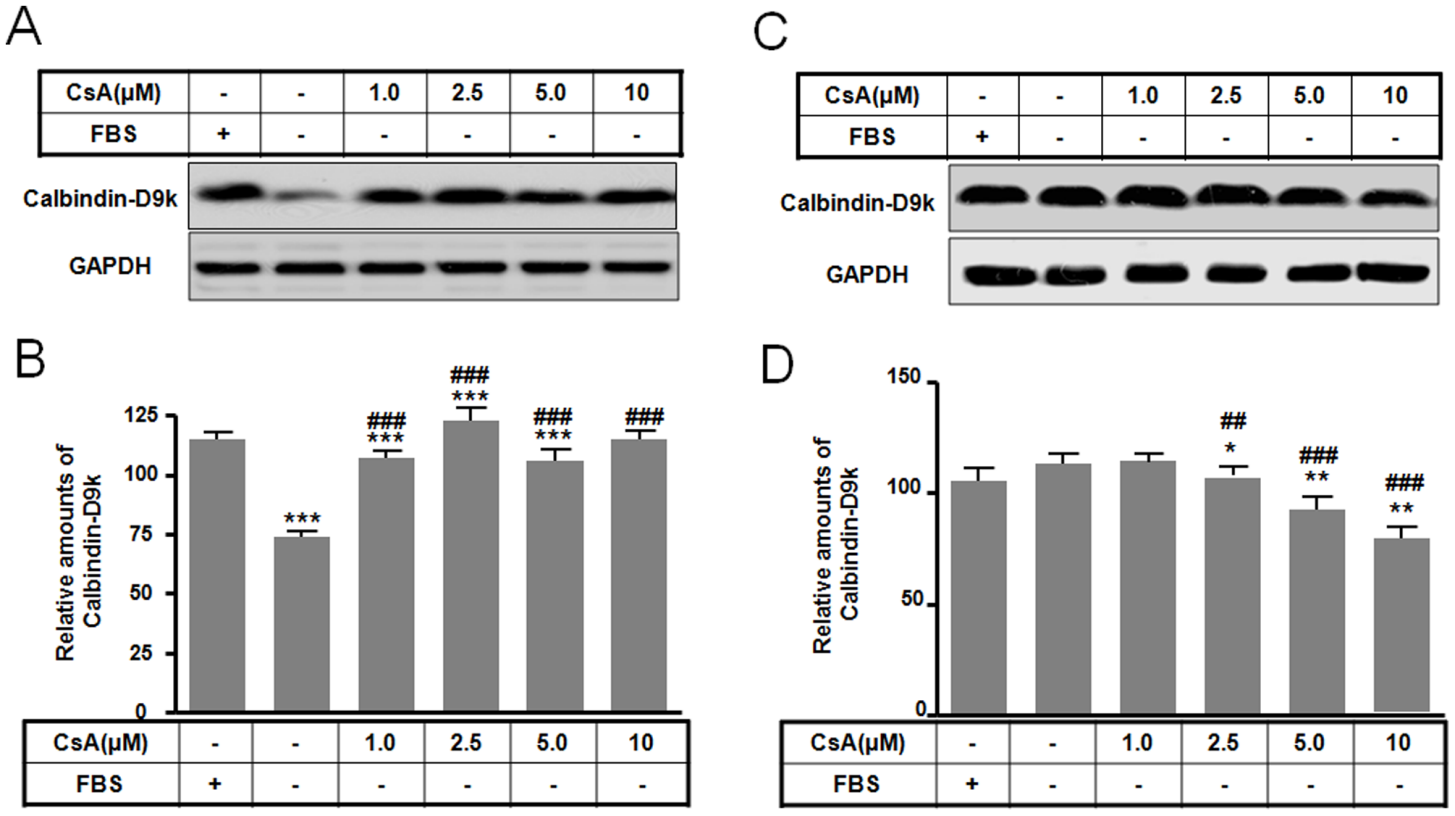
Effect of CsA-mediated autophagic and apoptotic death on calbindin-D9k levels. GH3 cells were incubated in DMEM with or without 10% fetal bovine serum in the presence or absence of CsA (0 to 10 µM) for 10 h. Calbindin-D9k levels for autophagy (A) and apoptosis (C) were determined by Western blotting and the relative amount of calbindin-D9k for autophagy (B) and apoptosis (D) was calculated as described in the Materials and Methods. *p<0.05, **p<0.01, ***p<0.001 vs. serum treatment. ^##^ *p*<0.01, ^###^ *p*<0.001 vs. no serum treatment.

In a recent report, Pallet et al. [13], [14] found that CsA induces ER stress in renal tubular cells. Indeed, the ER appears to be an initiator or a regulator of apoptosis [19]. Stimulation of inositol 1,4,5-triphosphate (IP3) causes Ca^2+^ release from the ER [20], which is involved in apoptotic signal transduction and is required for Ca^2+^-dependent DNA fragmentation [21]. To examine this pathway, we assayed the levels of the intracellular Ca^2+^ modulator calbindin-D9k. Calbindin-D9k levels were increased in autophagy compared to the serum-free control (Fig. 6A, B). However, calbindin-D9k levels were significantly decreased in apoptosis induced by 1.0 to 5 µM CsA (Fig. 6C, D). This result suggests that calbindin-D9k may be an important molecular determinant of apoptotic or autophagic cell death by CsA in rat pituitary GH3 cells.

## Discussion

CsA has been shown to induce autophagic and apoptotic cell death in both *in vivo* and *in vitro* experiments [12]–[14], [16], [17]. These researches suggest that the relative levels of ER stress responses may be a determinant of apoptotic cell death by depleting proapoptotic proteins. In other experiments, CsA induced apoptosis by up-regulating the proapoptotic factors p53 and Bax, cleaving PARP, and down-regulating the antiapoptotic factor Bcl-2 in cultured rat mesangial cells and in a rat chronic nephrotoxicity model [22], [23].

The mechanisms underlying chronic CsA nephropathy are not completely understood. Activation of the intrarenal renin-angiotensin system, increased release of endothelin-1, inappropriate apoptosis, stimulation of inflammatory mediators, and ER stress have all been implicated in the pathogenesis of chronic CsA nephropathy [10]. Pellet et al. [13] demonstrated that CsA induces autophagy in primary cultured human renal tubular cells through LC3-II expression and autophagosome visualization by electron microscopy. CsA-induced autophagy may occur downstream of ER stress. Various ER stresses activate autophagy, and salubrinal, an inhibitor of eIF2alpha dephosphorylation, both protects cells against ER stress and inhibits LC3-II expression.

The cross-talk between autophagic and apoptotic cell death pathways is complex [4], [5]. Three different types of interaction between these pathways have been postulated: (1) an apoptosis/autophagy partnership; (2) autophagy may antagonize apoptosis; or (3) autophagy may enable apoptosis. We demonstrated that CsA treatment increased nuclear fragmentation and Bax-2 levels, and reduced Bcl-2, resulting in apoptotic cell death. Translocation of Bcl-2 family members from the cytoplasm to the mitochondria is a central step in propagation of apoptotic signals to the cytoplasm [24]–[26]. In contrast, Bcl-2 levels increased during autophagic cell death. This result suggests that autophagy may act to interfere or to attenuate apoptosis and participate in cell protection. Autophagy was shown to be essential for survival during nutrient starvation *in vivo* and *in vitro* [27]–[30]. Autophagy also protected epithelial cells from apoptotic cell death [31] during metabolic stress [32], drug treatment, and radiation damage for genotoxic ROS [33].

Our data demonstrate that CsA activates p53 and triggers apoptotic cell death in GH3 cells. These data are consistent with other reports, which showed CsA treatment activates p53, induces nuclear localization, and activates the expression of the p53 target genes Bax, mdm2, and p21^waf1^ [24]. Importantly, inhibition of endogenous p53 or lack of functional p53 significantly reduces the extent of CsA-induced apoptosis, indicating that p53 is critical for CsA-mediated cell death. Our data show that an immunosuppressive drug is capable of activating p53 in GH3 cells.

Although a direct link between CsA-mediated ROS generation and adverse renal toxicity in rat renal tubular cells has not been demonstrated [34], it has been suggested that increased ROS levels may contribute to adverse CsA side effects [35], [36]. CsA-induced ROS generation and susceptibility to ROS are highly tissue-specific [37]. Autophagy is also regulated by ROS, including superoxide and hydrogen peroxide, though superoxide may be the central ROS that regulates autophagy [38], [39]. To examine the role of ROS in CsA mediated cell death, we examined ERK and SOD protein levels, components of the pathways that are activated in response to oxidative stress [40]. In addition, both phosphoinositide 3-kinase/protein kinase B (PI3K/PKB) and ERK pathways have been implicated in survival and death responses of mouse kidney cells [41]. CsA treatment of GH3 cells reduced Cu/Zn-SOD expression in autophagy (Fig. 5A, B) and Mn-SOD expression in apoptosis in a dose dependent fashion (Fig. 5D, F). These results suggest that the changes in Cu/Zn- and Mn-SOD expression are associated with apoptotic and autophagic cell death by CsA treatment in GH3 cells.

The ER appears to function as a key initiator or a regulator of apoptosis with Ca^2+^-mediated signaling [19], [42], An increase in mitochondrial matrix Ca^2+^ regulates metabolism, and Ca^2+^ also modulates mitochondrial permeability transition, which is controlled by permeability transition pores [43]. Permeability transition pores have been implicated in apoptotic, autophagic, and necrotic cell death pathways. We examined calbindin-D9k levels indirectly as a marker for intracellular Ca^2+^ modulation. Calbindin-D9k is expressed in the mammalian intestine (duodenum), kidney, pituitary gland, growth cartilage, bone, and female reproductive tissues [44]–[47]. Uterine calbindin-D9k has been shown to be involved in the regulation of myometrial activity by intracellular calcium [48]. Our results suggest that calbindin-D9k may act as an important intracellular calcium ion exchanger that regulates apoptotic or autophagic cell death by CsA in rat pituitary GH3 cells.

In summary, we found that CsA induces apoptotic and autophagic cell death in rat pituitary GH3 cells. Apoptotic and autophagic cell death could be distinguished both morphologically and molecularly as they displaced distinct levels of Bcl-2 and Mn-SOD expression.

## Materials and Methods

### Cell culture

Rat pituitary GH3 cells, which originated from the growth hormone-producing tumor of the rat anterior pituitary and somatomammotroph phenotype, were purchased from ATCC (CCL-82.1) and were cultured in Dulbecco's modified Eagle's medium (DMEM, GibcoBRL, Gaithersburg, MD, USA) supplemented with or without 10% heat-inactivated fetal bovine serum (GibcoBRL) at 37°C in 5% CO_2_ in a humidified atmosphere. Cells were treated with CsA (0 to 100 µM) in serum-free medium. For autophagy studies, culture dishes were coated with polyethyleneimine (20 µg/mL) (Sigma, St. Louis, MO, USA), washed with distilled water, and dried.

### Cell viability assays

Cell survival was quantified using Cell Counting Kit-8 (Dojindo Laboratories, Tokyo, Japan). In brief, GH3 cells were cultured in 96-well plates (Corning Inc., Corning, NY, USA) at a density of 5×10^3^ cells per well. The cells were cultured in the presence or absence of melatonin. After 15 h, cells were washed, treated with Cell Counting Kit-8 reagents, incubated in the dark for 4 h, and then absorbance (450 nm) was measured using a plate reader (Molecular Device, Sunnyvale, CA). Percent viability was calculated as the absorbance of the melatonin-treated sample/control absorbance ×100.

### Western blot analysis

Cells were harvested, washed two times with ice-cold PBS, and then resuspended in 20 mM Tris-HCl buffer (pH 7.4) containing a protease inhibitor mixture (0.1 mM phenylmethylsulfonyl fluoride, 5 µg/mL aprotinin, 5 µg/mL pepstatin A, 1 µg/mL chymostatin) and phosphatase inhibitors (5 mM Na_3_VO_4_, 5 mM NaF). Whole cell lysates were prepared with a Dounce homogenizer (20 strokes), followed by centrifugation at 13,000×g for 20 min at 4°C. Protein concentration was determined using a BCA assay (Sigma). Proteins (50 µg) were separated by 12% SDS-PAGE and then transferred onto polyvinylidene difluoride (PVDF) membranes. Membranes were hybridized with antibodies specific for LC3 (Santa Cruz Biotechnology, Santa Cruz, CA, USA), p53 (Santa Cruz Biotechnology), Cu/Zn- and Mn-SOD (Cell Signaling Technology, Beverly, MA, USA), p-ERK and ERK (Transduction Laboratories, Lexington, KY, USA), Bax and Bcl-2 (Santa Cruz Biotechnology), calbindin-D9k (Swant, Bellinzona, Switzerland), and GAPDH (Assay Designs, Ann Arbor, MI, USA). Immunoreactive proteins were visualized by exposure of the membrane to X-ray film. The films were scanned and the band intensities and optical densities were corrected by background subtraction, quantified using ImageJ analysis software (version 1.52, Wayne Rasband, NIH, Bethesda, MD, USA), and then normalized.

### Immunofluorescence staining

GH3 cells grown on culture slides (BD Falcon Labware, REF 354108) were permeabilized and fixed in methanol at −20°C for 3 min. Cells were washed with phosphate-buffered saline (PBS), blocked with 10% bovine serum albumin (Sigma) with PBS for 10 min, and incubated with primary antibody in blocking buffer for 1 h at room temperature (RT). Cells were hybridized with secondary antibodies for 1 h at RT. The coverslips were mounted on glass slides using Vectashield mounting medium (Vector Labs Inc., Burlingame, CA, USA). Cells were viewed under a Leica TCS SP5 confocal microscope (Leica, Microsystems CMS GmbH, Germany). The following primary antibodies were used: Lamp2 (Cell Signaling Technology) and LC3 (Cell Signaling Technology). The following secondary antibodies were used: Alexa 594 (red)-conjugated anti-rabbit IgG (Vector Laboratories Inc.) and fluorescein isothiocyanate (green)-labeled anti-mouse IgG (Jackson ImmunoResearch Laboratories, West Grove, PA, USA).

### Nuclear staining and immunofluorescence microscopy

Cells were fixed in 4% paraformaldehyde buffered with 0.1 M phosphate (pH 7.3) for 30 min and then washed with phosphate-buffered saline (PBS). Cells were permeabilized with 0.3% Triton X-100 for 20 min, washed with PBS, and then stained with 4,6-diamidino-2-phenylindole (DAPI, Santa Cruz, USA) for 10 min. For immunofluorescence microscopy, GH3 cells were grown on culture slides (Nunclon, Gibco cat. no. 176740, NV Invitrogen SA, Merelbeke, Belgium) and then fixed in cold methanol for 10 min at 20°C. Cells were washed in PBS, blocked with 5% bovine serum albumin in PBS for 30 min, and incubated with primary antibody (Bcl-2, Santa Cruz Biotechnology) in 2% bovine serum albumin (BSA) for 1 h at room temperature. Cells were washed with PBS and subsequently incubated with secondary antibody (fluorescein isothiocyanate (green)-labeled anti-mouse IgG (Jackson ImmunoResearch Laboratories) in 2% BSA for 1 h at room temperature. After washing with PBS, images were acquired at a peak excitation wavelength of 340 nm (OlympusIX71) using an OLYMPUS DP controller and manager (200× magnification).

### DNA fragmentation analysis

Cell pellets were resuspended in 750 µL of lysis buffer (20 mm Tris-HCl, 10 mm EDTA, and 0.5% Triton X-100, pH 8.0) and left on ice for 45 min with occasional shaking. DNA was extracted with phenol/chloroform and precipitated with alcohol. The precipitate was dried and resuspended in 100 µL of 20 mM Tris-HCl, pH 8.0. After degradation of RNA with RNase (0.1 mg/mL) at 37°C for 1 h, samples (15 µL) were electrophoresed on a 1.2% agarose gel in 450 mM Tris borate-EDTA buffer (TBE, pH 8.0) and photographed under UV light.

### Statistical analysis

Significant differences were detected by ANOVA, followed by Tukey's test for multiple comparisons. Analysis was performed using the Prism Graph Pad v4.0 (Graph Pad Software Inc., San Diego, CA, USA). Values are expressed as means ± SD of at least three separated experiments, in which case a representative experiment is depicted in the figures. *P* values<0.05 were considered statistically significant.
